# Plant growth and phytoactive alkaloid synthesis in kratom [*Mitragyna speciosa* (Korth.)] in response to varying radiance

**DOI:** 10.1371/journal.pone.0259326

**Published:** 2022-04-26

**Authors:** Mengzi Zhang, Abhisheak Sharma, Francisco León, Bonnie Avery, Roger Kjelgren, Christopher R. McCurdy, Brian J. Pearson

**Affiliations:** 1 Department of Environmental Horticulture, Mid-Florida Research and Education Center, Institute of Food and Agricultural Sciences, University of Florida, Apopka, Florida, United States of America; 2 Department of Pharmaceutics, College of Pharmacy, University of Florida, Gainesville, Florida, United States of America; 3 Department of Medicinal Chemistry, College of Pharmacy, University of Florida, Gainesville, Florida, United States of America; 4 Translational Drug Development Core, Clinical and Translational Science Institute, University of Florida, Gainesville, Florida, United States of America; Qingdao Agricultural University, CHINA

## Abstract

Leaves harvested from kratom [*Mitragyna speciosa* (Korth.)] have a history of use as a traditional ethnobotanical medicine to combat fatigue and improve work productivity in Southeast Asia. In recent years, increased interest in the application and use of kratom has emerged globally, including North America, for its potential application as an alternative source of medicine for pain management and opioid withdrawal syndrome mitigation. Although the chemistry and pharmacology of major kratom alkaloids, mitragynine and 7-hydroxymitragynine, are well documented, foundational information on the impact of plant production environment on growth and kratom alkaloids synthesis is unavailable. To directly address this need, kratom plant growth, leaf chlorophyll content, and alkaloid concentration were evaluated under three lighting conditions: field full sun (FLD-Sun), greenhouse unshaded (GH-Unshaded), and greenhouse shaded (GH-Shaded). Nine kratom alkaloids were quantified using an ultra-performance liquid chromatography-tandem mass spectrometry (UPLC-MS/MS) method. Greenhouse cultivation generally promoted kratom height and width extension by 93–114% and 53–57%, respectively, compared to FLD-Sun. Similarly, total leaf area and leaf number were increased by 118–160% and 54–80% under such conditions. Average leaf size of plants grown under GH-Shaded was 41 and 69% greater than GH-Unshaded and FLD-Sun, respectively; however, no differences were observed between GH-Unshaded and FLD-Sun treatments. At the termination of the study, total leaf chlorophyll *a+b* content of FLD-Sun was 17–23% less than those grown in the greenhouse. Total leaf dry mass was maximized when cultivated in the greenhouse and was 89–91% greater than in the field. Leaf content of four alkaloids to include speciociliatine, mitraphylline, corynantheidine, and isocorynantheidine were not significantly impacted by lighting conditions, whereas 7-hydroxymitragynine was below the lower limit of quantification across all treatments. However, mitragynine, paynantheine, and corynoxine concentration per leaf dry mass were increased by 40%, 35%, and 111%, respectively, when cultivated under GH-Shaded compared to FLD-Sun. Additionally, total alkaloid yield per plant was maximized and nearly tripled for several alkaloids when plants were cultivated under such conditions. Furthermore, rapid, non-destructive chlorophyll evaluation correlated well (r^2^ = 0.68) with extracted chlorophyll concentrations. Given these findings, production efforts where low-light conditions can be implemented are likely to maximize plant biomass and total leaf alkaloid production.

## Introduction

*Mitragyna speciosa*, commonly known as kratom, is a tropical small to medium size (4–16 m) tree indigenous to wetland forests of Southeast Asia. Historically, kratom was used in Thailand, Malaysia, and Indonesia to serve as a mild herbal stimulant, pain reliever, and to treat diarrhea and opium addiction [1–3]. Given its historical use as an analgesic and a medicine to mitigate opioid withdrawal symptoms, research on kratom cultivation and use is warranted. In Southeast Asia, kratom leaves are harvested and consumed fresh by chewing or steeping in water to make tea [3]. In the Western hemisphere where fresh kratom is unavailable, kratom is sold in the form of dried and ground powder or as a concentrated liquid extract for easier transportation and consumption [4].

Kratom produces an array of psychoactive compounds. So far more than 54 compounds including alkaloids, flavonoids, and terpenoids have been identified within kratom [5–7]. Although kratom’s alkaloids are likely produced by the plant to aid in defense of environmental challenges, they have demonstrated activity upon human central nervous system targets and may be medically valuable for the improvement of human health [8–10]. Of the wide array of alkaloids found in kratom leaves, mitragynine and 7-hydroxymitragynine are the best understood and considered the most psychoactive [6]. Mitragynine can constitute up to 38.7% in traditional and commercial kratom products [5, 11, 12]. 7-Hydroxymitragynine is produced by oxidation of mitragynine and is a minor constituent (< 0.01% in fresh leaves) found at concentrations of up to 2% in leaf extracts and commercial kratom products [13, 14]; however, it is believed to be the major contributor to the known addictive potential of kratom given its activity as a potent μ-opioid receptor agonist [15–18]. In the U.S., commercially available, imported kratom products (in the format of capsules, dried leaves, powders, resins, and concentrated extracts) have variable concentrations of mitragynine (1.2–38.74%) and 7-hydroxymitragynine (0.01–0.75%) on a weight basis *[11, 19]*. Other major and minor alkaloids found within leaves of kratom include paynantheine (0.3–12.8% of kratom dry leaf powder or extract weight), speciogynine (0.1–5.3%), mitraphylline which functions as muscle relaxants and possess anti-inflammatory properties, and speciociliatine (0.4–12.3%) and corynantheidine (0.1–1.2%), which act as opioid agonists and adrenergic receptor [6, 10, 20, 21]. The overall effect following consumption of kratom leaves is complex due to the interplay and range of bioactive alkaloids present [22].

Despite kratom’s long history of use in Southeast Asia, information on factors that influence plant growth and alkaloidal synthesis are largely unavailable. Available research on kratom is largely focused on its leaf chemistry and its potential pharmacological applications. As interest in cultivation of kratom increases along with consumptive demand, formal kratom cultivation efforts will likely soon be established. Although kratom had long been cultivated in Thailand, it was made illegal in 1943, and thus planting, possession, sales, and use of kratom leaves were prohibited [23]. In 2019, the Thai government approved a bill legalizing kratom for medicinal applications while recreational use remains illegal [24]. This bill provides the first opportunity for legal cultivation of kratom in Thailand since passage of the Kratom Act of 1943 and the Narcotics Act of 1979. Similar to other agricultural production efforts, plant cultivation practices based upon empirical evidence will be necessary for consistent successful commercial cultivation of kratom.

Biosynthesis of phytoactive leaf alkaloids can occur in response to light intensity. Highest natural photosynthetic light intensity, or photosynthetic photon flux density (PPFD), occurs outdoors in full-sun conditions during summer in the northern hemisphere. Plants respond to high PPFD by upregulating or downregulating alkaloid synthesis dependent upon species and other environmental factors present. For example, camptothecin, an indole alkaloid in *Camptotheca acuminate* leaves, was reduced by 99% when plants were moved from full sun to heavy shade (27% full sun) [25]. Similarly, total alkaloid content in tubers of *Pinellia ternate* decreased 27% when plants were moved from full sun to heavy shade (15% full sun) [26]. Light intensity in combination with nutrient availability may collectively affect phytoactive alkaloid production in some plant species. Winters and Loustalot [27] observed limited synthesis of alkaloids in roots of *Cinchona ledgeriana* seedlings when subjected to 30% of full sunlight across a range of nitrogen fertility regimes. However, considerable alkaloid synthesis did occur when light intensity was high and availability of nitrogen was low.

Phytoactive alkaloid synthesis within leaves can be affected by light quality, especially ultraviolet (UV) and far-red light, although studies are limited. Light quality is defined as the spectral composition of wavelengths influential to plant growth and photosynthesis. Concentration of the alkaloids catharanthine and vindoline in cell suspension culture of *Catharanthus roseu*s was promoted 3- and 118-fold, respectively, after being exposed to UV-B irradiation for a duration of 48 h as compared to plants not exposed to UV-B [28]. In addition to the influence of UV light, red and far-red light can influence plant secondary metabolism responsible for synthesis of leaf alkaloids. A low red to far-red light ratio, which occurs naturally in high shade or dense canopy conditions, results in a low phytochrome stationary state that can cause induction of shade avoidance responses, such as internode elongation and increase of leaf area and leaf chlorophyll concentration to optimize photosynthesis efficiency in the presence of competing vegetation [29–31]. However, the impact of light quality on alkaloid synthesis is still relatively unclear and largely undocumented. Tso et al. [32] observed that total alkaloid content in tobacco (*Nicotiana tabacum*) tended to be higher in plants that were subjected to end-of-day red than far-red radiation; however, differences were not significant. Although wild populations of kratom have been documented in the dense equatorial rain forests of Thailand and Malaysia, the influence of light on growth and alkaloid synthesis is undocumented and is vital to future commercial kratom cultivation efforts.

Although wild populations of kratom are found in the dense understory of equatorial rain forests, open-canopy commercial farming has recently been established in Indonesia in response to high export demands [33]. The influence of high light on plant growth and alkaloid content under open canopy production conditions, however, is undocumented. To determine if light-induced environmental factors can modify or influence synthesis of kratom leaf alkaloids, a preliminary investigation was conducted where kratom trees cultivated in a greenhouse were sampled to quantify leaf alkaloid content, moved to an outdoor, full-sun environment, and then sampled again two weeks later [34]. Concentrations of mitragynine slightly increased, although not significantly, in response to the change in cultivation environment, thus suggesting that an increase in PPFD, an increase in air temperature, a change in light quality, or a combination of these environmental factors may be influential to synthesis of alkaloids within leaves of kratom.

Given these preliminary findings, coupled with increased demand for kratom and a lack of foundational information regarding its cultivation, empirically derived information is needed by growers and producers to assist in attainment of biomass yield and alkaloid production goals. To directly address this need, research was conducted to examine the influence of light on kratom: 1) growth, leaf area, and biomass, 2) leaf chlorophyll content and its estimation through a rapid, non-destructive technique, and 3) the concentration of nine leaf alkaloids. Research results provide an expanded foundational knowledge of kratom and its response to varying light environments. This information will be helpful to the newly emerging commercial kratom cultivation industry where optimization of operations will be key to efficient and predictable production.

## Materials and methods

### Plant materials

Vegetative propagules, or cuttings, were taken from a single mother stock kratom plant, treated with 1000 mg·L^-1^ indole-3-butyric acid rooting hormone (Hormodin 1, OHP Inc., Mainland, PA, United States), and then placed within rockwool cubes to develop roots. Once roots had visually emerged from the rockwool cubes, the propagules were transplanted and cultivated in 0.7 L and 11.4 L containers as described by Zhang et al. [4]. Osmocote Plus 15-9-12 slow-release fertilizer (Scotts, Marysville, OH, United States) was applied at 74 g per container as per the manufacturer’s recommendations to provide sufficient nutrient availability throughout the duration of the experiment.

### Experiment treatments

Sixty plants (n = 60) were randomly assigned to one of three diverse light treatments to include: direct full sun in the field (FLD-Sun), unshaded within a greenhouse (GH-Unshaded), and shaded within a greenhouse (GH-Shaded) in Apopka, Florida, United States (lat. 28°38’ N, long. 81°33’ W). Plants within FLD-Sun were placed under direct full sunlight outside of the greenhouse in an open space field. Plants within the GH-Unshaded treatment were placed onto a bench within an enclosed greenhouse to receive ambient solar radiation. Lastly, plants within the GH-Shaded treatment were placed onto a bench inside of the greenhouse under shade cloth. Polycarbonate glazing materials reduced the daily light integral within the greenhouse (GH-Unshaded) by approximately 60% compared to FLD-Sun. A knitted shade cloth (DeWitt, Sikeston, MO) installed approximately 2 m above a greenhouse bench reduced light by another 40% (~25% of full sun) to create conditions for the GH-Shaded treatment. Environmental conditions within treatment areas were measured and adjusted to ensure limited variability existed. All plants were grown under natural day length regardless of treatment.

### Environmental conditions

Plant irrigation schedule and greenhouse environment were monitored as described by Zhang et al. [4]. Fafard 4P (Sun Gro Horticulture Canada Ltd., Agawam, MA, United States) containing 48% Canadian sphagnum peat, 30% pine bark, 10% perlite, and 12% vermiculite with a pH of 5.5–6.5 was used as soilless substrate. Outdoor environmental conditions were recorded every 15 min by the onsite Florida Automated Weather Network station. Average temperature within the greenhouse and the field were relatively similar throughout the experiment, with a mean of 27.7–28.1°C in September, 24.3–25.0°C in October, 19.9–21.7°C in November, and 17.4–18.9°C in December 2018.

Data collection protocol for this research was described previously in detail by Zhang et al. [4]. Briefly, plant height, width, trunk diameter, and SPAD value (an index of relative chlorophyll concentration) of mature leaves were collected monthly beginning September 10, 2018. Total leaf number and area, average leaf size, and total leaf dry mass was recorded at termination of the experiment on December 20, 2018. Specific leaf area was calculated by total leaf area and leaf dry mass. Quantification of leaf alkaloids and chlorophyll *a*, *b*, and *a+b* concentration was conducted monthly using the methods described in Zhang et al. [4]. In brief, leaf chlorophyll content was extracted and measured with a UV-Visible Spectrophotometer from three random plants within each treatment once every four weeks. A multiple reaction mode (MRM) based UPLC-MS/MS method in positive ionization was implemented for the quantification of nine kratom alkaloids on Acquity Class I UPLC coupled with Waters Xevo TQ-S Micro triple quadrupole mass spectrometer. UPLC method, compound and source parameters were the same as reported previously [4].

### Experiment design and data analysis

The experiment was conducted using a complete randomized design with three treatments and 20 replicates. Each plant was considered as an experimental unit and an individual leaf sample was considered a subsample within the experimental unit. Statistical analysis was conducted using a restricted maximum likelihood mixed model analysis in JMP® Pro 13 (SAS Institute, Inc., Cary, NC, United States) and SAS (SAS Institute, Inc., Cary, NC, United States). Post-hoc mean separation tests were performed using Tukey’s honest significant difference test by lighting treatment with treatment combination replicates (n = 20) defined as the random error term. Statistical tests were considered significant if *P* < 0.05.

## Results

### Plant growth indicators

**Fig 1:**
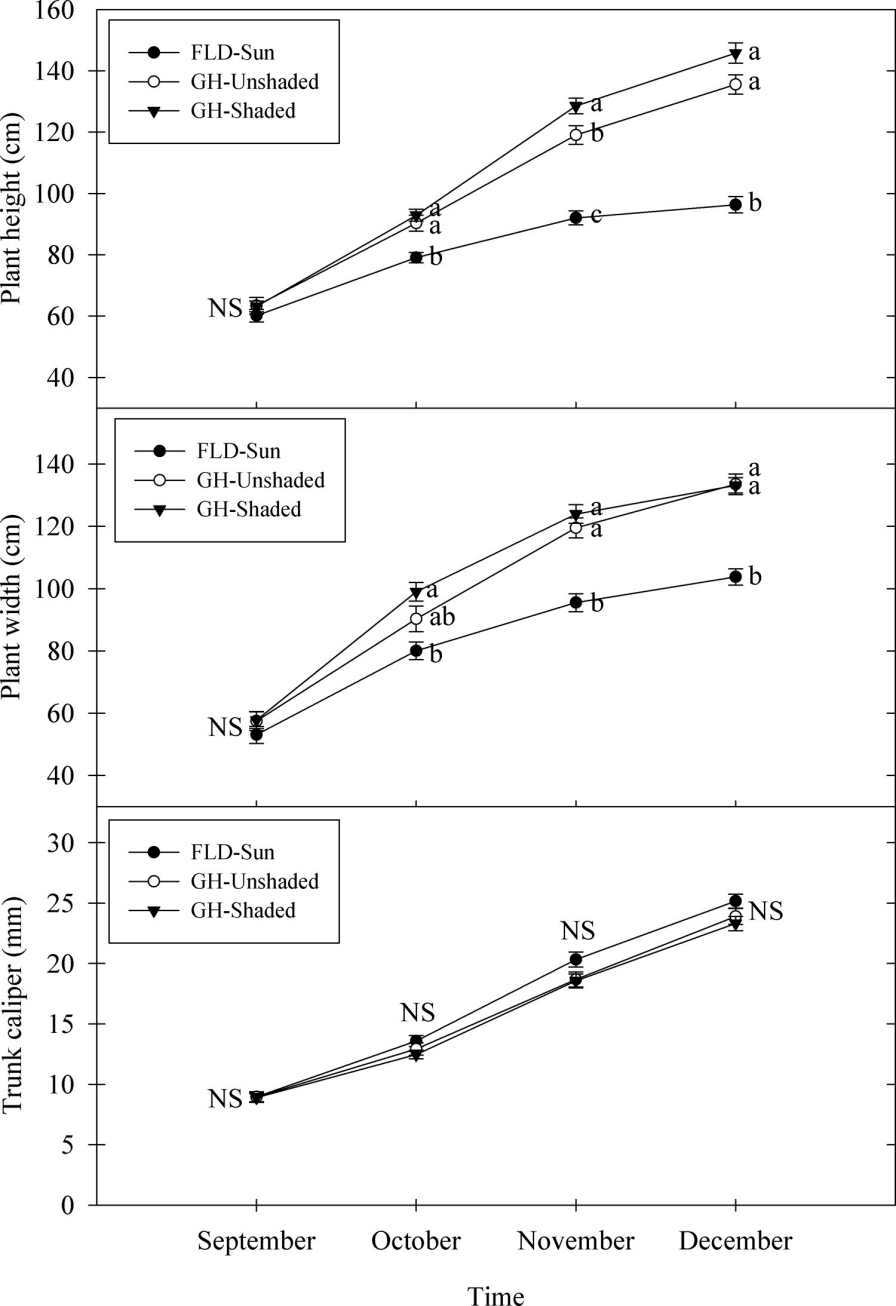
Plant height, width, and trunk caliper of kratom cultivated under varying radiance. FLD = field; GH = greenhouse. Data were pooled from twenty replicates per treatment (n = 60) each month. Means sharing the same letter are not statistically different by Tukey’s honest significant difference test at *P* < *0*.*05*. Error bars indicate the standard error. NS = not significant.

**Fig 2:**
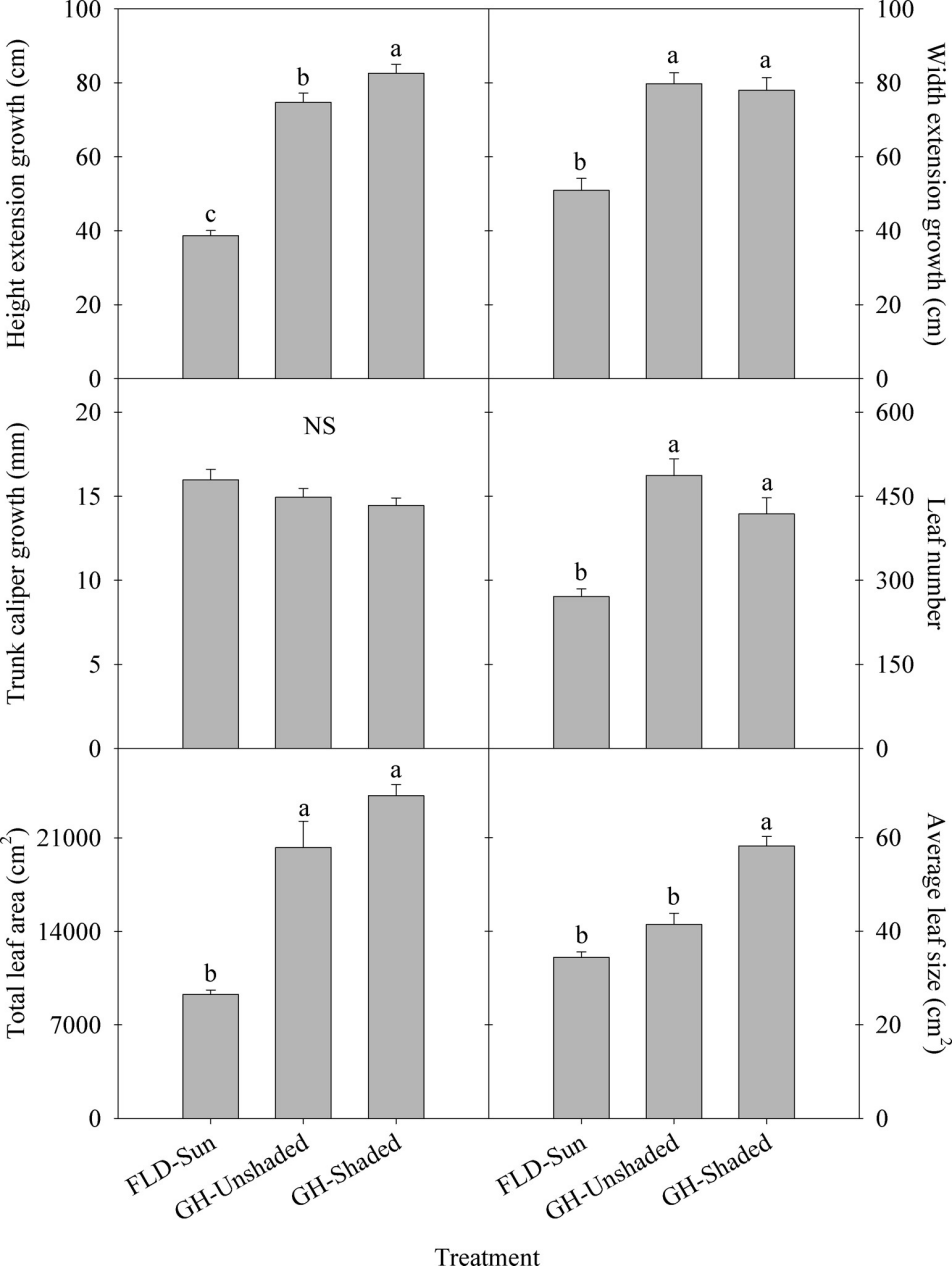
Average plant growth indicators of kratom cultivated over time under varying radiance. FLD = field; GH = greenhouse. Leaf number included leaves ≥ 2 cm. Data were pooled from twenty replicates per treatment (n = 60) for height, width, and trunk caliper growth and four random replicates per treatment (n = 12) for leaf number, total leaf area and average leaf size. Means sharing the same letter are not statistically different by Tukey’s honest significant difference test at *P* < *0*.*05*. Error bars indicate the standard error. NS = not significant.

Plants had similar height, width, and trunk caliper at the initiation of the experiment (Fig 1). As time progressed, plants grew taller and wider in the greenhouse compared to FLD-Sun (Fig 1). Height extension of plants cultivated within the greenhouse over time was greater than FLD-Sun, with the tallest plants resulting from GH-Shaded (Figs 1 and 2). Plant height extension was increased by 93 and 114% in response to GH-Unshaded and GH-Shaded, respectively, compared to FLD-Sun (Fig 2). Similar to height extension, plant width extension of those grown in the greenhouse were between 53 and 57% greater than FLD-Sun. Despite differences in height and width in response to imposed light treatments, trunk caliper growth of plants was similar among all treatments overtime (Figs 1 and 2).

**Fig 3:**
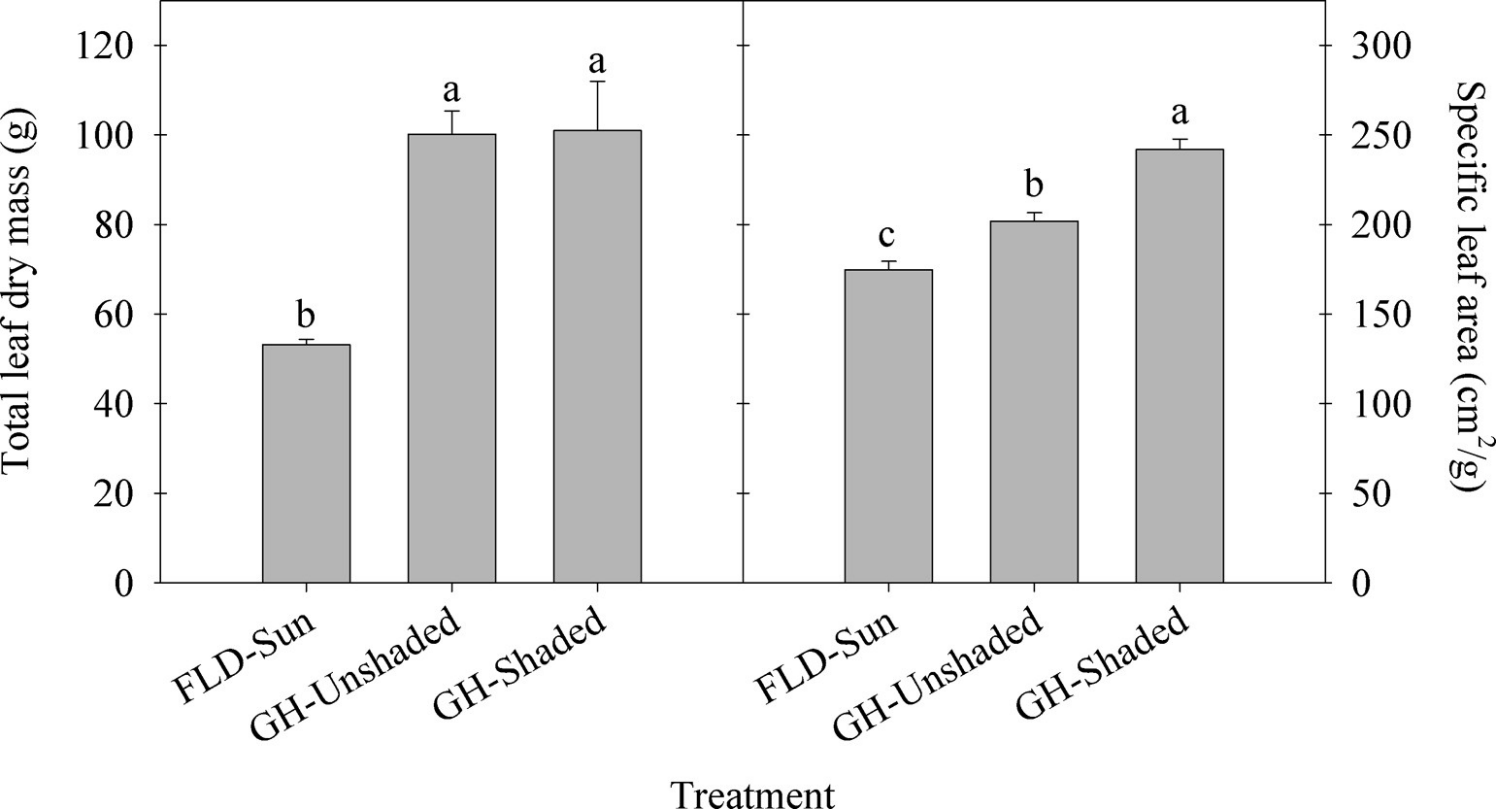
Total leaf dry mass and specific leaf area of kratom cultivated under varying radiance. FLD = field; GH = greenhouse. Data were pooled from four random replicates per treatment (n = 12) and means sharing the same letter are not statistically different by Tukey’s honest significant difference test at *P* < *0*.*05*. Error bars indicate the standard error.

Total leaf area and the number of leaves on plants grown inside the greenhouse regardless of shading were statistically similar and were 118–160% and 54–80% greater, respectively, than FLD-Sun (Fig 2). Average leaf size was similar between FLD-Sun and GH-Unshaded. However, GH-Shaded had between 41 and 69% greater average leaf size than GH-Unshaded and FLD-Sun, respectively (Fig 2). Total leaf dry mass trends were similar to that observed for total leaf area, plant width, and height, with greenhouse cultivated kratom having 89–91% greater total leaf dry mass than FLD-Sun (Fig 3). Additionally, specific leaf area increased with the decrease of light received, at 16 and 39% higher under GH-Unshaded and GH-Shaded, respectively, compared to FLD-Sun.

### Chlorophyll concentration and SPAD

**Fig 4:**
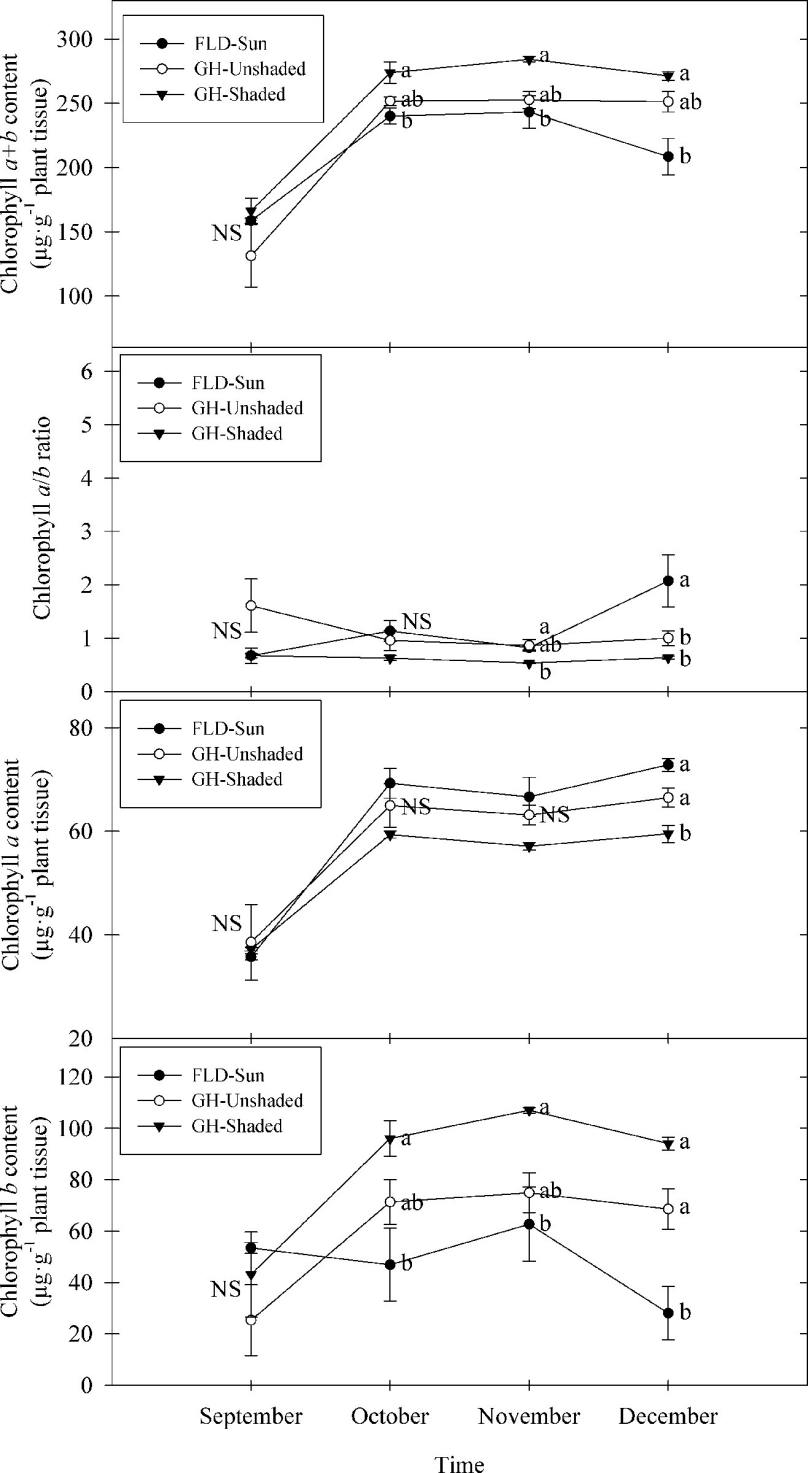
Chlorophyll *a*, *b*, *a*+*b* content, and chlorophyll *a*/*b* ratio of kratom cultivated under varying radiance. FLD = field; GH = greenhouse. Data were pooled from three random replicates per treatment for four months (n = 36). Means sharing the same letter are not statistically different by Tukey’s honest significant difference test at *P* < *0*.*05*. Error bars indicate the standard error. NS = not significant.

**Fig 5:**
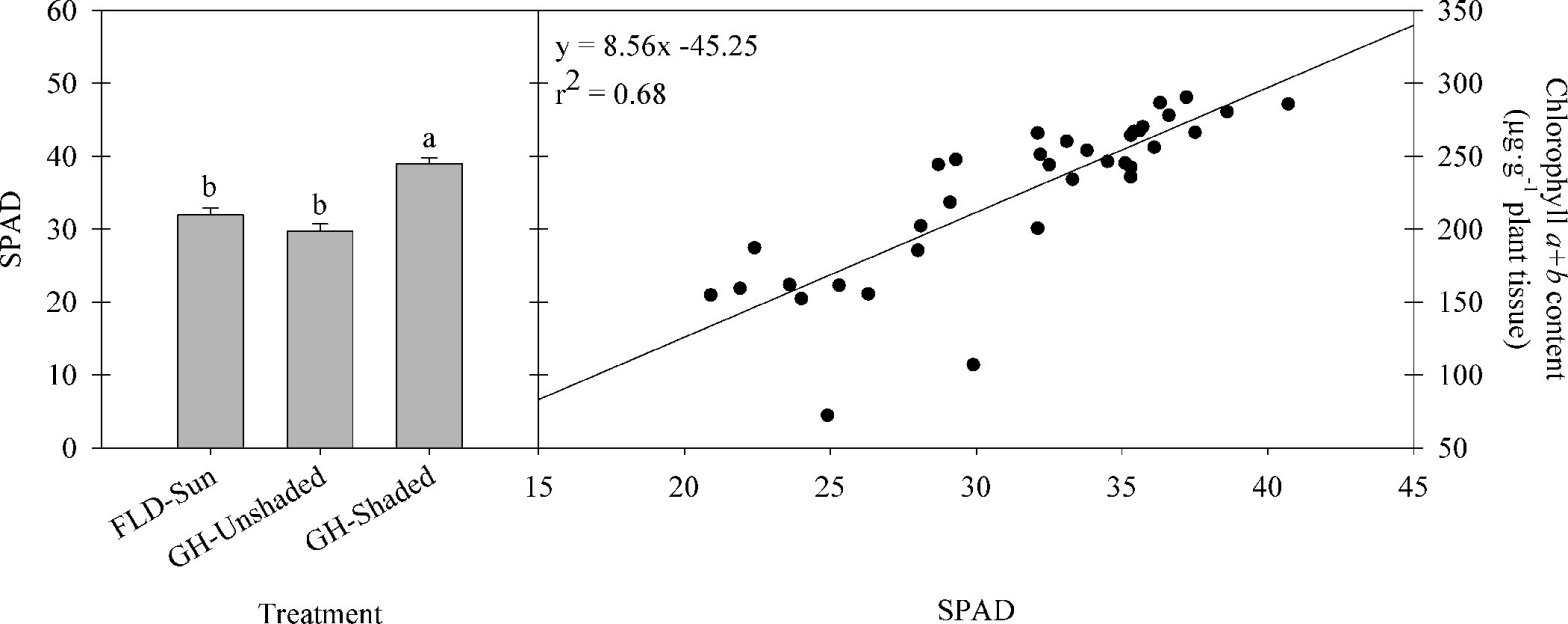
SPAD index value and correlation of SPAD and chlorophyll *a+b* content in kratom leaves. SPAD index measurements were pooled from four random replicates per treatment for four months (n = 48) and chlorophyll correlation data were pooled from nine random replicates for four months (n = 36). Plants were cultivated from September to December 2018 under different radiation treatments. Means sharing the same letter are not statically different by Tukey’s honest significant difference test at *P* < *0*.*05*. Error bars indicate the standard error.

Individual chlorophyll *a*, chlorophyll *b*, and total chlorophyll *a+b* content among treatments were similar at the beginning of the experiment and generally increased during the first month, with a more rapid increase in GH-Shaded, followed by GH-Unshaded and FLD-Sun (Fig 4). No significant differences among treatments were observed in chlorophyll *a* for the first three months; however, chlorophyll *a* of GH-Shaded was significantly lower than the other two treatments by 11–18% in the last month of the experiment. Conversely, chlorophyll *b* showed significant differences among treatments by the second month, with GH-Shaded being the highest, followed by GH-Unshaded and FLD-Sun, with the same trend followed through the experiment (Fig 4). At the termination of the experiment, the total leaf chlorophyll *a+b* content of FLD-Sun was 17–23% less than those grown inside the greenhouse, but no differences were found between greenhouse treatments. On the contrary, chlorophyll *a/b* ratio of FLD-Sun was greatest and was more than double that of plants grown in the greenhouse. Additionally, SPAD values were 22–31% greater in GH-Shaded than GH-Unshaded or FLD-Sun, respectively (Fig 5). SPAD values correlated well (r^2^ = 0.68) with chlorophyll concentrations among trees between September and December 2018 (Fig 5).

### Alkaloid concentration

7-hydroxymitragynine was not detected in any of our samples (Table 1). Mitragynine was detected in 53% of the samples, with concentrations 31–40% higher under GH-Shaded compared to GH-Unshaded or FLD-Sun on a per leaf dry mass basis (Table 1). Similarly, paynantheine concentrations in GH-Shaded were 35 and 23% greater than FLD-Sun and GH-Unshaded, respectively. Corynoxine concentrations were approximately 2-fold greater in GH-Shaded compared to the other two treatments. On the contrary, speciogynine achieved the highest concentration under FLD-Sun and was 23–24% greater than plants cultivated in the greenhouse. Interestingly, although not significant, speciociliatine concentration of GH-Unshaded was slightly higher than the other two treatments. Despite these trends, differences in concentrations of mitraphylline, corynantheidine, and isocorynantheidine in response to lighting treatments were not observed.

**Table 1:** Phytoactive alkaloidal content per leaf dry mass (±se) and total alkaloidal content per plant (±se) grown under direct sunlight in the field (FLD-Sun) or in greenhouse (GH) unshaded or shaded.

Treatment Alkaloid	Alkaloid concentration per leaf dry mass (%w/w)			Total alkaloid content per plant (g)		
	FLD-Sun	GH-Unshaded	GH-Shaded	FLD-Sun	GH-Unshaded	GH-Shaded
Mitragynine	0.015±0.001 b	0.016±0.001 b	0.021±0.001 a	0.79±0.04 c	1.60±0.14 b	2.10±0.11 a
7-Hydroxymitragynine	Below LLOQ*	Below LLOQ	Below LLOQ	Below LLOQ	Below LLOQ	Below LLOQ
Speciogynine	0.135±0.008 a	0.112±0.006 b	0.113±0.006 ab	7.35±0.44 b	11.28±0.64 a	11.41±0.59 a
Paynantheine	0.020±0.001 b	0.022±0.001 b	0.027±0.001 a	1.04±0.04 c	2.23±0.13 b	2.83±0.16 a
Speciociliatine	0.021±0.001	0.024±0.002	0.023±0.001	1.12±0.06 b	2.64±0.18 a	2.37±0.14 a
Mitraphylline	0.142±0.008	0.119±0.007	0.123±0.007	7.77±0.46 b	12.05±0.71 a	12.37±0.65 a
Corynantheidine	0.084±0.006	0.080±0.007	0.068±0.007	4.68±0.36 b	7.38±0.57 a	5.57±0.41 b
Isocorynantheidine	0.103±0.005	0.098±0.007	0.094±0.007	5.48±0.28 b	9.48±0.66 a	9.48±0.71 a
Corynoxine	0.018±0.001b	0.018±0.002 b	0.038±0.003 a	0.97±0.06 b	1.79±0.17 b	3.86±0.31 a

Data were pooled from four replicates bi-weekly for four months. Means sharing the same letter are not statistically different by Tukey’s honest significant difference test at *P* < *0*.*05*.

*LLOQ = Lower Limit of Quantification (0.01%w/w).

On a per plant basis, GH-Shaded drastically promoted total mitragynine synthesis (~2.7-fold greater than FLD-Sun) (Table 1). Similarly, total paynantheine content was 2.7- and 2.1-fold greater under GH-Shaded and GH-Unshaded, respectively, than FLD-Sun. Total content of corynantheidine was maximized when grown in GH-Unshaded and was 58 and 32% greater than plants grown under FLD-Sun and GH-Shaded, respectively. Corynoxine content per plant achieved the highest concentration under GH-Shaded and was 116–298% greater than the other treatments. Additionally, total speciogynine, speciociliatine, mitraphylline, and isocorynantheidine content of plants grown in the greenhouse, regardless of shading conditions, were approximately 1.5- to 2.4-fold greater compared to FLD-Sun.

## Discussion

Various species exhibit shade-acclimation response when subjected to shade or a low red to far-red light ratio environment to maximize sunlight interception [35–37]. In our study, kratom height, average leaf size, and total leaf dry mass were increased in response to unshaded and shaded conditions in the greenhouse compared to plants grown under full sun outdoors. Plants also developed significantly more specific leaf area and total chlorophyll content with a reduction in the chlorophyll *a*/*b* ratio as the irradiance decreased. This suggested an optimization of light capture and a higher efficiency of light use in response to maximize photosynthesis and gain carbon under shaded conditions [38, 39]. Similar findings have been observed in other related studies. For example, the height and leaf area of poinsettia (*Euphorbia pulcherrima*) were 55–75% and 111–155% greater when grown under 48–78% shading compared to 30% shading [40]. Additionally, total chlorophyll content was highest under 92% of shade and dry weight was greatest under 48% of shade. In a separate study, plant height of four different species of Pacific Northwest conifer seedlings were greatest and chlorophyll *a* was consistently higher under 75% of shade compared to no shade [41]. These shade-acclimation changes likely assist kratom in being more competitive in the dense, light-limited tropical forests in which they evolved and provide evidence of shade acclimation response within this species.

Although chlorophyll *a* is the primary pigment involved in plant photosynthesis process, chlorophyll *b* availability and synthesis strongly regulates the ultimate accumulation of light harvesting complexes and the importation of the proteins for both Photosystem I and II [42, 43]. In our study, GH-Shaded plants achieved the highest total chlorophyll content primarily due to the significant increase in chlorophyll *b*. A lower chlorophyll *a*/*b* ratio in GH-Shaded kratom is consistent with plant response to shade in related studies [44, 45]. This trend, together with plant growth and biomass accumulation (Figs 1 and 2), suggested kratom optimized its photosynthetic efficiency when cultivated in low light growing conditions. We believe that kratom is a shade tolerant species as it exhibited shade tolerant characteristics including but not limited to an increased total chlorophyll content and a decreased chlorophyll *a*/*b* ratio under shade conditions. Additionally, given that the total leaf chlorophyll content was reliably estimated using SPAD meter values and fit a linear model developed in our study, cultivators of kratom may choose to reliably predict chlorophyll content using the rapid, nondestructive technique offered by the SPAD device.

Although originally believed to be byproducts of plant primary metabolic processes, phytoactive alkaloids are now better understood to be purposefully produced by plants to protect against herbivory and disease [9]. Relationships between environmental stimuli and the regulation of alkaloidal synthesis are complex and highly variable among plants and environments. In our study, mitragynine, paynantheine, and corynoxine had the highest concentration per leaf dry mass under the most shaded conditions. This is supported by Ralphs et al. [46] where short-term shade stress, induced by 30% full sunlight for three days using shade cloth and 100% of full sunlight by covering leaves with aluminum foil, increased alkaloid concentration in tall larkspur (*Delphinium barbeyi*) by 36–38% and 11%, respectively, compared to plants grown in open sun. Similarly, several plant alkaloids including vinblastine (from *Catharanthus roseus*) and camptothecin (from *Camptotheca acuminata*) have been reported in higher concentrations following exposure to low light conditions [25, 47]. However, contradictory relationships were observed in speciogynine within our study. The specific synthesis pathway of speciogynine, paynantheine, as well as other bioactive alkaloids, are not fully understood within kratom; however, the interrelatedness of the structures of a wide array of kratom indole and oxindole alkaloids, as described by Flores-Bocanegra et al. [7], may explain some observed trends. Presumably, both speciogynine and paynantheine are downstream metabolites of mitragynine, and the increase of paynantheine at least partially attribute to the decrease of speciogynine. Although thought to be an overly simplified relationship, our findings support the carbon/nutrient balance theory where carbon stress due to limitation of light and a resulting reduction in photosynthesis increase nitrogen-containing defense compounds, such as alkaloids, in shade-tolerant species [48]. Additionally, a high specific leaf area for plants growing in shade can make their leaves more sensitive to mechanical stress and herbivory, thus increased alkaloid levels may assist with the defense mechanism for survival in deep shade [38, 45, 49].

Cultivating kratom under shade cloth within a greenhouse maximized the concentration of mitragynine, paynantheine, and corynoxine. In addition, this same production condition maximized plant height, leaf area, and leaf size. Given this, total calculated yield of each alkaloid quantified in our study was greatest among shaded plants given the shade acclimation response of greater leaf mass and a larger leaf size (Table 1). More specifically, GH-Shaded conditions promoted mitragynine concentration by 40% per leaf dry mass and almost tripled the total mitragynine alkaloid yield per plant. An evolutionary adaptation to low-light environments is likely given the higher plant performance observed when kratom was cultivated in conditions similar to those that occur in the dense, shaded understory of equatorial rainforests. Despite the range of lighting conditions imposed in this study, no 7-hydroxymitragynine was detected in any leaf samples, suggesting low abuse liability potential when compared to previously examined imported commercial kratom product and reinforcing the opinion that this alkaloid is produced from mitragynine as a post-harvest artifact [5].

Although historically regarded as a field crop, data from our study indicated that greenhouse production of kratom may be economically valuable given significant increased alkaloid concentrations and greater total alkaloid yield. Alternatively, field-erected shade structures may be effective in providing sufficient shade for increased leaf alkaloid concentrations while offering a relatively low production cost. Given that individual alkaloids responded differently to the cultivation environments imposed, additional research is needed to fully understand the medicinal impacts of shade-cultivated kratom.

When conducting horticultural investigations, differences in irradiance usually accompany a differential in environmental temperature; however, environmental temperature information is often not discussed, reported, or otherwise accounted for in available literature. In our study, greenhouse and field temperatures were managed so they remained similar throughout the experiment and thus variable temperatures among imposed treatments were eliminated as a potential confounding variable. Research examining synthesis of leaf alkaloids in response to different temperatures under similar light intensities in the controlled environments is warranted.

In addition to being influenced by light intensity and temperature, synthesis of phytoactive alkaloids may be influenced by light quality. Indole alkaloid concentrations have been observed to vary in response to UV-B irradiation exposure in a number of medicinal plants including *Clematis terniflora*, *Withania somnifera*, *Coleus forskohlii*, *Zanthoxylum bungeanum*, and *Coleus aromaticus* [50]. Ultraviolet light transmission is often 20–80% lower within greenhouses than outdoors in full sun, dependent upon glazing materials used in the construction and design of the greenhouse [51]. Surprisingly, most alkaloid concentrations, except speciogynine, in our study were not different between plants cultivated under FLD-Sun and GH-Unshaded. In a previous preliminary study [34], a slight but not significant increase of mitragynine was observed after plants were moved from within the greenhouse to outdoors. Given that the preliminary study was only exploratory, plant number and experiment duration were limited, and the environment was not strictly controlled, we believe that this observation may have been caused by plant individual differences and not a light treatment effect. The preliminary study also relied upon a different analytical method to quantify alkaloid concentrations, thus confounding accurate comparisons between studies. Moreover, differences may have been due to increased aging of greenhouse materials and degradation of the UV stabilizer found in its roof material. Thus, more UV radiation entered the greenhouse in this study and created little to no UV difference compared to the field [52].

Significant differences in the concentration of the alkaloids mitragynine, paynantheine, and corynoxine were observed among plants subjected to GH-Shaded and GH-Unshaded conditions. In addition to reducing light intensity, shade cloth modifies light quality by causing a shift in the red to far-red light ratio. Together, results suggested that a change of light intensity, a change of light quality, or a combination of both may result in the alteration of leaf phytoactive alkaloids in kratom, particularly in the case of corynoxine where concentrations differed more than 2-fold in response to lighting treatments. Future research on alkaloid synthesis in response to different light quality remains essential.

## Conclusion

Given recent increased interest in the cultivation and application of kratom, foundational research examining the influence of light on kratom growth and alkaloid synthesis was conducted. Lighting conditions significantly influenced plant growth and the synthesis of leaf alkaloids. Mitragynine, paynantheine, and corynoxine concentrations, per leaf dry mass, were maximized under shade conditions when cultivated within a greenhouse. Moreover, low-light conditions significantly promoted plant growth and increased total leaf dry mass and thus, as a result, drastically enhanced alkaloid yield per plant. Given these findings, production efforts where low-light conditions can be implemented would be recommended to maximize plant biomass and total alkaloid leaf concentrations.
